# Expression and functional analysis of the TatD-like DNase of *Plasmodium knowlesi*

**DOI:** 10.1186/s13071-018-3251-4

**Published:** 2018-12-12

**Authors:** Yapan Zhou, Bo Xiao, Ning Jiang, Xiaoyu Sang, Na Yang, Ying Feng, Lubin Jiang, Qijun Chen

**Affiliations:** 1 Key Laboratory of Zoonosis, Shenyang Agricultural University, Dongling Road 120, Shenyang, 110866 China; 2 Unit of Human Parasite Molecular and Cell Biology, Key Laboratory of Molecular Virology and Immunology, Pasteur Institute of Shanghai, Chinese Academy of Sciences, Shanghai, 200031 China; 3 University of Chinese Academy of Sciences, Beijing, 100049 China

**Keywords:** *Plasmodium knowlesi*, TatD-like DNase, Surface, DNA hydrolysis, Mg^2+^, Virulence factor

## Abstract

**Background:**

In recent years, human infection by the simian malaria parasite *Plasmodium knowlesi* has increased in Southeast Asia, leading to growing concerns regarding the cross-species spread of the parasite. Consequently, a deeper understanding of the biology of *P. knowlesi* is necessary in order to develop tools for control of the emerging disease. TatD-like DNase expressed at the surface of *P. falciparum* has recently been shown to counteract host innate immunity and is thus a potential malaria vaccine candidate.

**Methods:**

The expression of the TatD DNase of *P. knowlesi* (PkTatD) was confirmed by both Western-blot and immunofluorescent assay. The DNA catalytic function of the PkTatD was confirmed by digestion of DNA with the recombinant PkTatD protein in the presence of various irons.

**Results:**

In the present study, we investigated the expression of the homologous DNase in *P. knowlesi*. The expression of TatD-like DNase in *P. knowslesi* (PkTatD) was verified by Western blot and indirect immunofluorescence assays. Like that of the *P. falciparum* parasite, PkTatD was also found to be located on the surface of erythrocytes infected by the parasites. Biochemical analysis indicated that PkTatD can hydrolyze DNA and this activity is magnesium-dependent.

**Conclusions:**

We identified that PkTatD expressed on the surface of *P. knowlesi*-infected RBCs is a Mg^2+^-dependent DNase and exhibits a stronger hydrolytic capacity than TatD from *P. falciparum*. The data support our previous findings that TatD-like DNase is a unanimously expressed virulence factor of *Plasmodium* parasites.

## Background

The simian malaria parasite *Plasmodium knowlesi* is the pathogen of a neglected tropical disease. *P. knowlesi* infection progresses rapidly and causes high parasitemia, which has severe consequences [1]. However, although it can cause severe and occasionally even fatal diseases in humans, it is seldom classified as a regular infectious agent by public health bodies [2]. *Plasmosium knowlesi* was first described in 1932 by Das Gupta and Knowles. Until 1965, it was considered as a zoonosis. Furthermore, until 2004, a proportion of natural *P. knowlesi* infections acquired by human patients in Sarawak, Malaysia, were likely misdiagnosed as being caused by the morphologically similar parasite *P. malariae* [3, 4]*.* Since then, the development of nested polymerase chain reaction [5] and dual-color fluorescence *in situ* hybridization assay [6] techniques as new diagnostic tools has significantly increased the accuracy of *P. knowlesi* detection. This has led to an increase in the identification of human *P. knowlesi* malaria in other parts of Malaysia, revealing that *P. knowlesi* is now the main cause of malaria in Malaysia [7]. Extensive study has revealed that *P. knowlesi* is widespread not only in Malaysia but in other countries in Southeast Asia, such as Singapore [8], Cambodia [9], Indonesia [10], Thailand [11], the Philippines [12] and Vietnam [2, 13]. Furthermore, imported *P. knowlesi* infections due to forest ecotourism in Southeast Asia have been reported in Europe and Japan [14]. Consequently, *P. knowlesi* is now recognized as the fifth *Plasmodium* parasite that infects humans, the others being *P. falciparum*, *P. malariae*, *P. vivax* and *P. ovale* [15].

Natural immunity mediated by immune cells such as macrophages, neutrophils and natural killer cells plays an important role in the elimination of invading pathogens from a host. For example, neutrophils provide an early response to inflammatory reactions, whereupon they are activated by chemotactic signals and then migrate quickly to infection sites [16]. However, clinical pathological investigations have revealed that the infiltration of innate immune cells to sites where *Plasmodium* parasites accumulate is not very significant. This is largely due to countermeasures employed antagonistically by such parasites against the host’s immune system, as outlined below.

During pathogen infection, activated neutrophils and macrophages release fibrous elements composed of proteases and DNA to restrict the invading pathogens [17, 18]. The fibrous elements released by neutrophils are termed as neutrophil extracellular traps (NETs) and they facilitate the innate immune response by capturing pathogens [19, 20]. However, *P. falciparum* and *Streptococcus* release DNase to counteract these NETs [21].

Thus, inhibition of pathogen-derived DNases can impede the propagation of pathogens in the host and help the immune system to control the infection. Furthermore, the TatD-like DNase of *P. falciparum* has been characterized as an important virulence factor and a potential malaria vaccine candidate [22, 23].

Herein, we reveal that *P. knowlesi* also expresses a TatD-like DNase (PkTatD), homologous to that of *P. falciparum*, and that it has a similar molecular structure and functionality.

## Results and discussion

### Sequence characteristics of the *P. knowlesi* TatD-like DNase

**Fig. 1:**
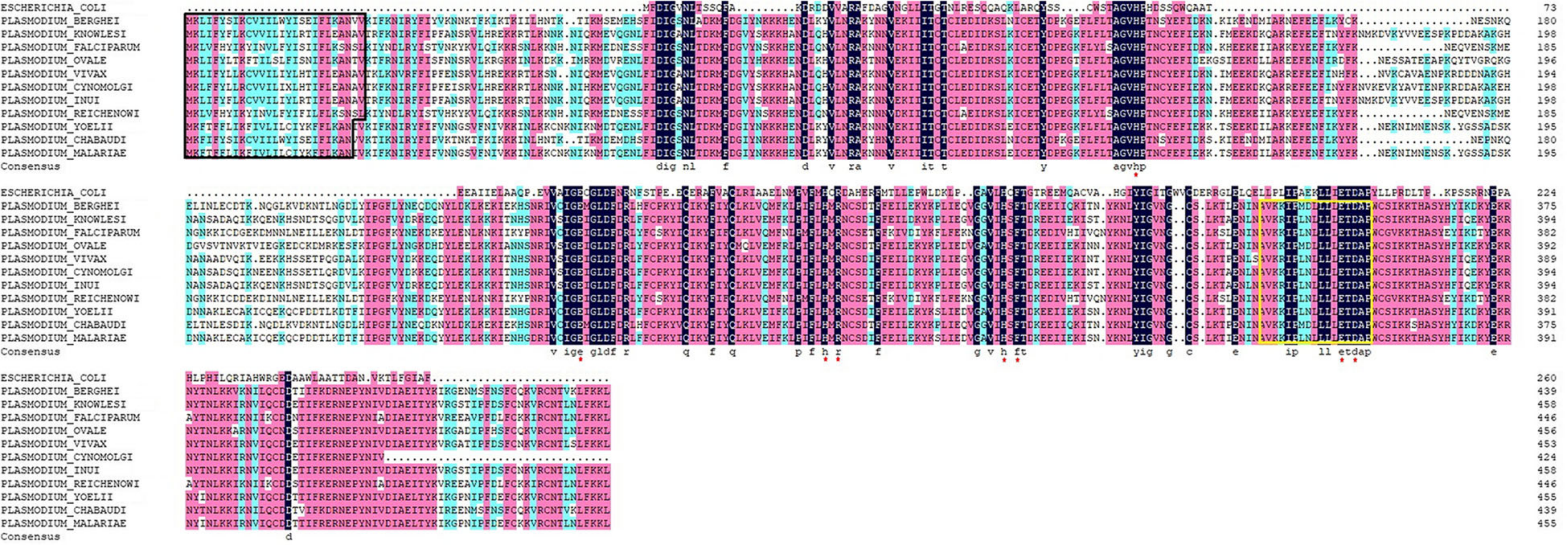
Sequence alignment of *Plasmodium* TatD proteins. The alignments of TatD sequences of *Escherichia coli* and *Plasmodium* species show that the plasmodial TatD sequences contain a 25–27AAs signal peptide (black box). All *Plasmodium* species have a conserved motif of 17 AAs (yellow box). The sequence similarity among *Plasmodium* species is up to 73.01%. The red asterisks indicate conserved active site residues

Sequence analysis of *Plasmodium* TatD proteins showed that TatD proteins are conserved in *Plasmodium* spp. As shown in Fig. 1, sequence similarity of TatD sequences among *Plasmodium* spp. was up to 73.01% and all TatD sequences contain a conserved motif with 17 amino acids typical to the TatD deoxyribonuclease family; this suggests that the TatD family proteins of *Plasmodium* parasites perform a similar DNA catalytic function in the interaction with their hosts*.* Furthermore, a signal peptide consisting of 25 amino acids was located at the N-terminus, which indicates, unlike that of other organisms [24], that the *Plasmodium* TatD proteins are likely secreted outside the infected cells.

### Preparation of PkTatD recombinant proteins and anti-PkTatD polyclonal antibodies

**Fig. 2:**
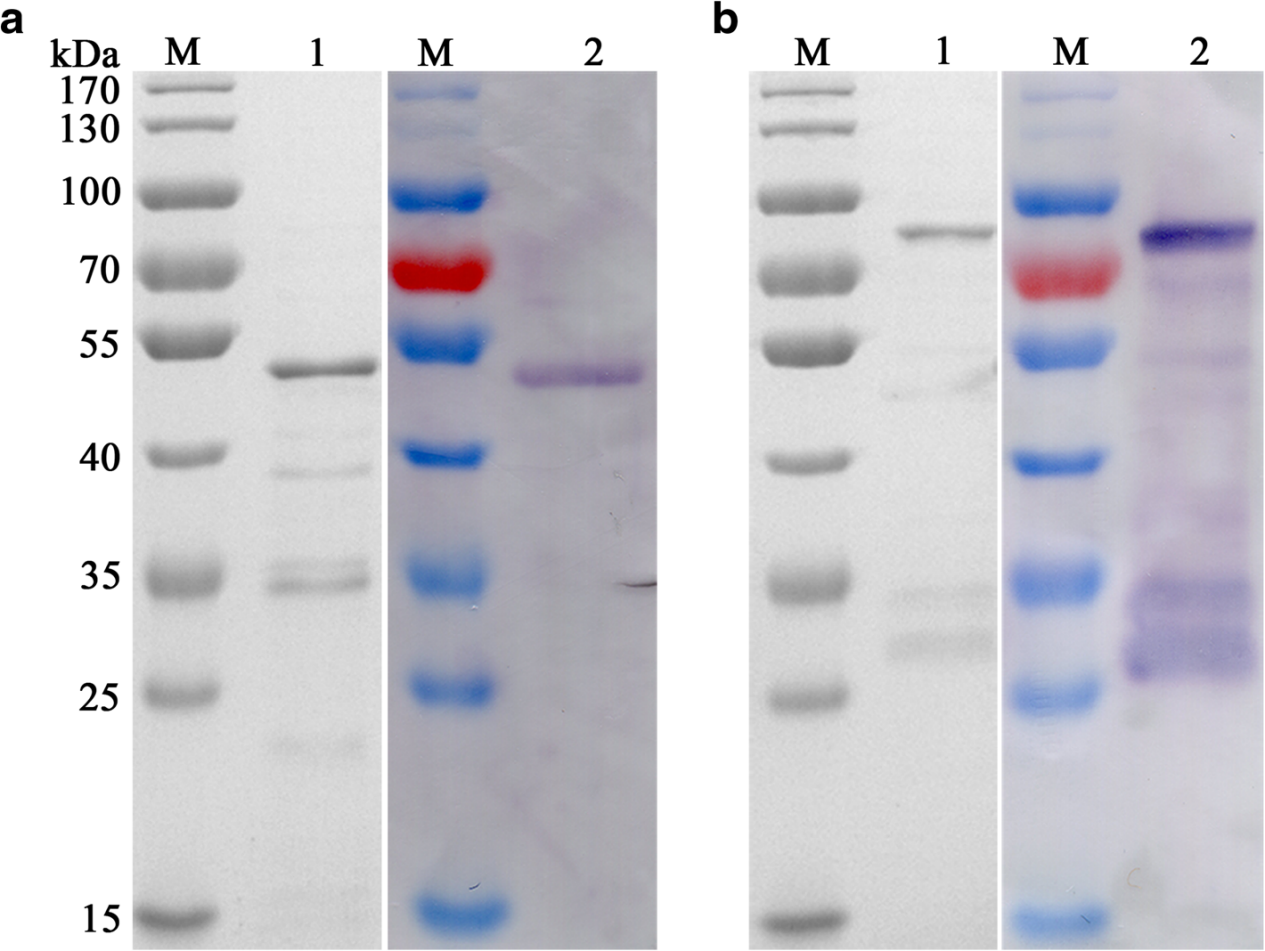
Generation and purification of soluble recombinant proteins. **a** The purified His-tagged recombinant protein (51.8 kDa). **b** The purified GST-tagged recombinant protein (76.8 kDa). Lane M: protein molecular weight markers; Lanes 1 and 2: SDS-PAGE and Western blot analyses, respectively

For the functional characterization of the PkTatD, recombinant proteins and specific antibodies were essentially generated. The optimized expression conditions for the recombinant proteins were found to be 22 °C, 16 h, after induction with 0.1 mM IPTG. The molecular weights of the His-tagged and GST-tagged recombinant proteins, without the putative signal peptide, were 51.8 and 76.8 kDa, respectively. The recombinant proteins were verified by Western blot using anti-His-tagged and anti-GST-tagged monoclonal antibodies, respectively. The soluble His-tagged (Fig. 2a) and GST-tagged (Fig. 2b) recombinant proteins were purified by affinity chromatography and verified by Western blot. The purified His-tagged recombinant protein was used to prepare the polyclonal antibodies. Both recombinant PkTatD proteins and specific antibodies are essential tools for further characterization of the molecule.

### Identification of endogenous TatD-like DNase in *P. knowlesi*

**Fig. 3:**
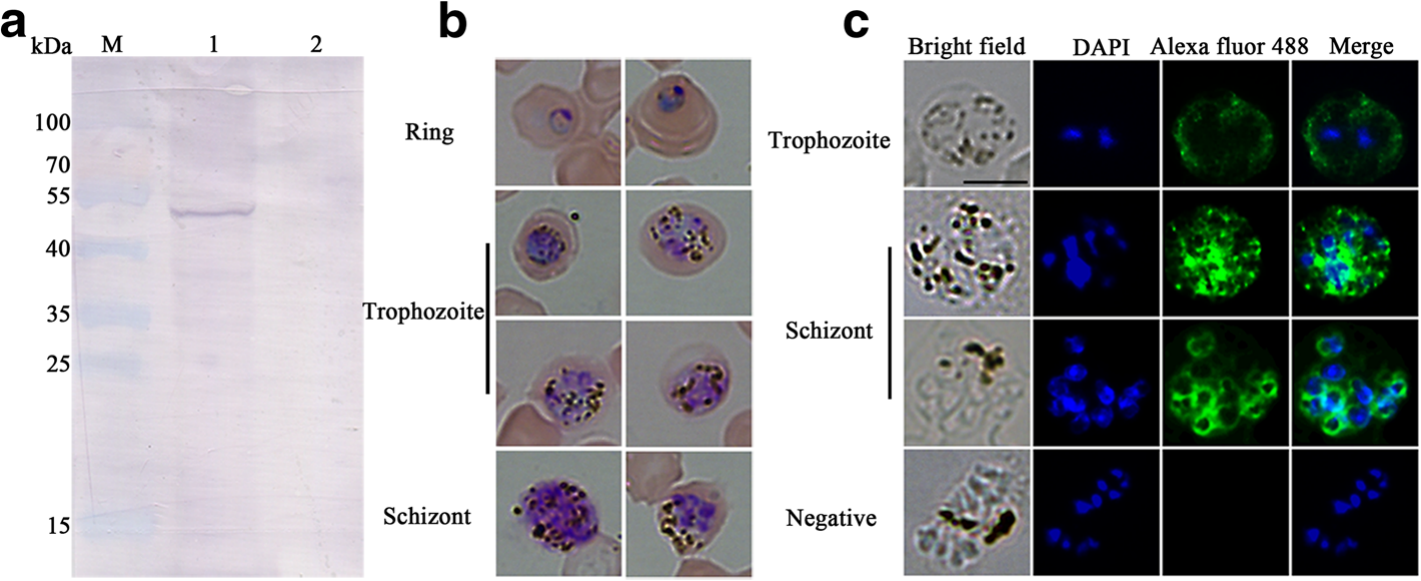
Identification of endogenous TatD protein in *P. knowlesi*. **a** Western blot analysis of the expression of native PkTatD. Lanes 1 and 2 represent infected RBCs and healthy macaque RBCs (negative control), respectively. A single band of approximately 54 kDa was detected in infected RBCs. **b** The different stages of *P. knowlesi* were stained with Giemsa. **c** Indirect immunofluorescence assay with anti-PkTatD polyclonal antibodies. The green fluorescence indicates that the PkTatD is located on the surface of the infected RBCs and in the cytoplasm of the parasite. The nuclei were stained blue with DAPI. *Scale-bar*: 5 μm

The expression of PkTatD in *P. knowlesi* was initially verified by Western blot using an anti-PkTatD antibody. The anti-PkTatD antibody reacted with a single 54 kDa protein, which is consistent with the molecular weight of PkTatD, and the PkTatD-specific antibody did not react with macaque RBCs (Fig. 3a). Furthermore, the results of IFA indicated that PkTatD is mainly located on the surfaces of the parasite-infected RBCs and in the cytoplasm of the infected erythrocytes (Fig. 3b, c), which was in a similar pattern to that of erythrocytic stage *P. falciparum* [22].

**Fig. 4:**
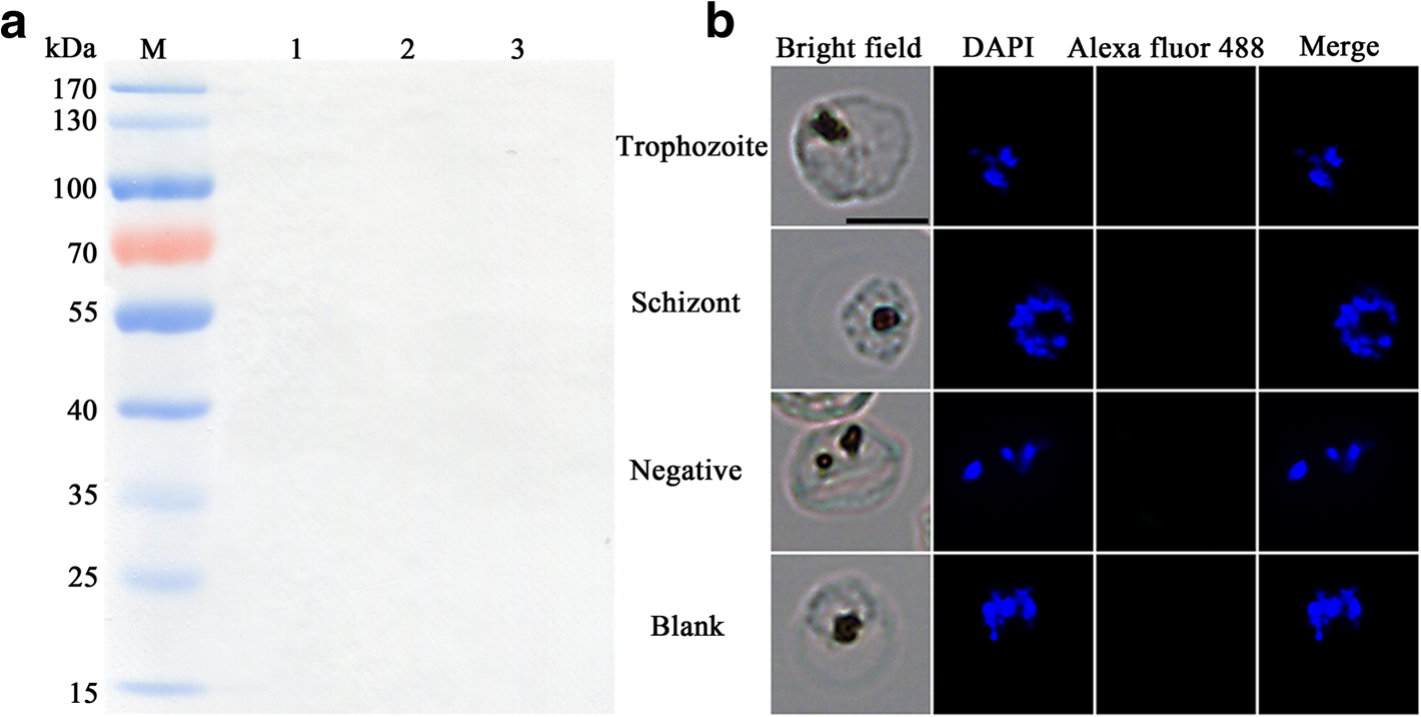
Anti-PkTatD-like DNase polyclonal antibodies did not cross react with that of *P. falciparum*. The cross reactivity of anti-PkTatD-like DNase antibodies with PfTatD-like DNase was tested with Western blot (**a**) and immunofluorescence (**b**), and no cross reactivity was observed. The parasite nuclei were stained blue with DAPI. Negative means a serum from a healthy rat was used as a negative control. Blank means no primary antibody was added in the first step incubation. *Scale-bar*: 5 μm

Even though the sequence similarity between PfTatD and PkTatD is 63.70%, the *P. knowlesi* TatD-like DNase specific antibody did not cross-react with *P. falciparum* TatD (PfTatD) in both Western blot (Fig. 4a) and IFA (Fig. 4b), suggesting that the immune epitopes of the two molecules are different and the antibodies to either species are likely not cross-protective.

### Divalent-metal dependence of PkTatD activity

**Fig. 5:**
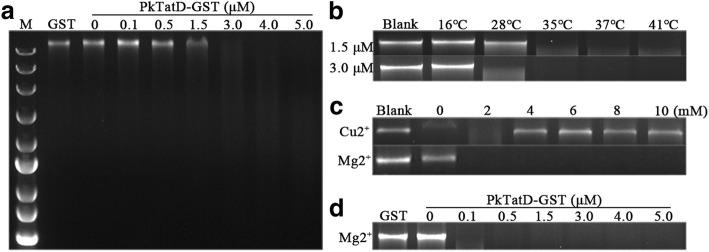
Ion-dependent DNase activity of PkTatD. **a** PkTatD-GST recombinant protein at concentrations from 0 to 5 μM was incubated with 200 ng of DNA in PBS (pH 7.4) at 37 °C for 1 h. DNA was completely hydrolyzed at a concentration of 3 μM. GST protein was used as a negative control. **b** DNA was completely hydrolyzed at 35 °C and the enzyme remained active at 41 °C. **c** PkTatD-GST recombinant protein and DNA were incubated with divalent metal ions at concentrations from 0 to 10 mM. The enzyme activity was enhanced by Mg^2+^ but inhibited by Cu^2+^. **d** Mg^2+^ (10 mM) and DNA were incubated with recombinant PkTatD-GST protein at concentrations from 0 to 5 μM

The DNA hydrolysis activity of *P. falciparum* TatD-like DNase has been previously characterized [22]. Here, we demonstrated that by incubating PkTatD GST-tagged recombinant protein with DNA and different concentrations of various divalent metal ions, PkTatD also hydrolyzed DNA in a concentration-dependent manner (Fig. 5a). Similar to that of PfTatD, the catalytic activity of PkTatD increased at higher temperatures (Fig. 5b). However, we found that the DNA hydrolytic activity of PkTatD is Mg^2+^-dependent (Fig. 5c, d), and higher concentrations of Cu^2+^ was inhibitory to the enzyme (Fig. 5c).

Deoxyribonucleases generally fall into two categories: divalent metal ion-dependent DNase I and divalent metal ion repressed DNase II [25, 26]. It has been previously reported that the activity of Yeast TatD is Mg^2+^-dependent [27], but the activity of *P. falciparum* TatD is inhibited by Mg^2+^ [22]. In this study, the hydrolysis activity was strongly enhanced when Mg^2+^ was added (Fig. 5c, d), but inhibited by Cu^2+^ (Fig. 5c). Thus, PfTatD showed a biochemical feature of DNase II, while PkTatD likely belonged to the DNase I group.

## Conclusions

We have identified a TatD-like DNase in *P. knowlesi*, PkTatD, which shared a conserved sequence feature with other *Plasmodium* species and was expressed both on the surface of *P. knowlesi*-infected RBCs and inside the cells. PkTatD is a Mg^2+^-dependent DNase and exhibits a stronger hydrolytic capacity that PfTatD. The data reported here further demonstrate that TatD DNases are essential factors for the plasmodial parasites in their interaction with the host immune system.

## Methods

### Bioinformatic analysis of *P. knowlesi* TatD-like DNase

The putative TatD sequence alignments of *P. knowlesi*, *P. berghei*, *P. falciparum*, *P. ovale*, *P. vivax*, *P. cynomolgi*, *P. inui*, *P. reichenowi*, *P. yoelii*, *P. chabaudi*, *P. malariae* and *E. coli* were retrieved from the PlasmoDB database (www.plasmodb.org) and signatures of the sequences were bioinformatically analyzed using DNAMAN 7 (Lynnon Corporation, San Ramon, USA).

### Parasite culture

The *P. falciparum* strain 3D7 was cultured using human O^+^ erythrocytes in malaria culture medium (MCM) according to standard methods [28]. The parasites were synchronized with 5% sorbitol.

The *P. knowlesi* A1-H1 strain was cultivated as previously described [29]. It was originally isolated from a human traveler returning from Malaysia in 1965, most likely from a zoonotic infection, and has since been passaged through rhesus macaque monkeys and subsequently adapted *in vitro* in rhesus macaque RBCs. The RBCs were provided by the Zoological Research Center of Chinese Academy of Sciences. Briefly, parasites were proliferated in macaque erythrocytes and MCM and then synchronized by centrifugation through a cushion of Nycodenz (Axis-Shield, Oslo, Norway) [30].

### Expression and purification of PkTatD recombinant proteins

The coding sequence of the PkTatD gene (PKNH_0201600) was optimized for expression in *E. coli* by synthesis and cloned into the pGEX4T-1 and pET28a vectors. His-tagged and GST-tagged recombinant proteins were expressed in *E.coli* (TransGen Biotech, Beijing, China) and the soluble proteins were purified by affinity chromatography using glutathione sepharose and His GraviTrap (GE Healthcare, Uppsala, Sweden), respectively [31].

### Preparation of anti-PkTatD polyclonal antibodies

Five female Wister rats were immunized with a total of 200 μg/rat His-tagged recombinant protein emulsified in complete Freund’s adjuvant (Sigma, Missouri, USA) (for the first immunization) and in incomplete Freund’s adjuvant (for the next three immunizations). The immune sera were collected after the antibody titer reached 1:16,000.

### Detection of PkTatD by Western blot assay

The expression of PkTatD protein of synchronized *P. knowlesi* trophozoites was analyzed through SDS-PAGE and a Western blot assay. The trophozoites and schizonts of *P. falciparum* were also analyzed using the same sera to assess the cross-reactivity of the anti-PkTatD antibodies. Briefly, the denatured proteins were electrophoresed in a 10% acrylamide gel then transferred onto PVDF membranes. The membranes were blocked for 2 h with 3% BSA and incubated overnight at 4 °C with the serum (1:200) from an infected rat as the primary antibody (the serum from a healthy rat was used as a control). After washing in 1× PBS buffer, the membranes were incubated with an alkaline-phosphatase-labeled goat anti-rat IgG antibody (1:20,000) for 1 h. Thereafter, the membranes were developed in a buffer containing BCIP/NBT (5-bromo-4-chloro-3-indolyl phosphate/nitroblue tetrazolium chloride).

### Indirect immunofluorescence assay

The indirect immunofluorescence assay (IFA) technique was used to further localize the PkTatD protein in the infected erythrocytes. Thin smears of *P. knowlesi*-infected and *P. falciparum*-infected erythrocytes were respectively fixed in cold methanol for 10 s and blocked with 3% BSA at 37 °C for 30 min. The samples were then incubated for 1 h at 37 °C with the immune sera (1:50) as primary antibodies (serum from a healthy rat was used as a control), then incubated with Alexa Fluor 488 goat anti-rat IgG (1:200) for 1 h at 37 °C. The parasite nuclei were stained with DAPI. The images were captured with a fluorescence microscope (Leica Camera AG, Wetzlar, Germany).

### Analysis of the DNA hydrolytic activity of the PkTatD recombinant protein

The genomic DNA of a mouse liver was extracted by phenol/chloroform extraction. The DNA was incubated with GST-tagged recombinant PkTatD protein at a concentration of 0.1, 0.5, 1.5, 3, 4 or 5 μM in PBS buffer (pH 7.4) and a total volume of 20 μl at 37 °C. The GST protein and PBS were used as negative control and blank control, respectively. To identify the optimum enzymatic temperature, the hydrolysis reaction was carried out at 16 °C, 28 °C, 35 °C, 37 °C and 41 °C. To investigate the effects of divalent metal ions on the enzyme activity, different concentrations of Mg^2+^, Cu^2+^, and other divalent metal ions were added to the reactions. The results were analyzed using 1.2% agarose gel.

## Abbreviations

BCIP/NBT: 5-bromo-4-chloro-3-indolyl phosphate/nitroblue tetrazolium chloride
IPTG: isopropyl-β-D-thiogalactoside
MCM: malaria culture medium
PBS: phosphate-buffered saline
PfTatD: *Plasmodium falciparum* TatD-like DNase
PkTatD: *Plasmodium knowlesi* TatD-like DNase
SDS-PAGE: sodium dodecyl sulfate polyacrylamide gel electrophoresis
